# Understanding the Link Between Discrete Negative Emotions and Empathic Accuracy

**DOI:** 10.1093/geroni/igab046.1281

**Published:** 2021-12-17

**Authors:** Cornelia Wieck, Denis Gerstorf, Oliver Schilling, Martin Katzorreck, Anna Lücke, Ute Kunzmann

**Affiliations:** 1 Leipzig University, Leipzig, Sachsen, Germany; 2 Humboldt University of Berlin, Humboldt University of Berlin, Brandenburg, Germany; 3 University of Heidelberg, Heidelberg, Baden-Wurttemberg, Germany; 4 University of Leipzig, Leipzig, Sachsen, Germany; 5 Heidelberg University, Heidelberg, Baden-Wurttemberg, Germany

## Abstract

Extant theories have suggested that negative emotions generally harm cognitive processes. However, adopting a discrete emotion perspective, in this study, we predicted that only anger and fear but not sadness should be negatively associated with empathic accuracy, a process that has been shown to be cognitively highly demanding. Over 100 participants (Mage = 66.66 years, SDage = 1.00) reported their emotional reactions in response to a negative film in the laboratory, documented their everyday momentary emotions six times a day over seven consecutive days, and completed a film-based empathic accuracy test. Initial findings suggest that only fear but not anger or sadness was related to empathic accuracy. More specifically, high levels of fear both in the laboratory and in everyday life predicted low empathic accuracy. This pattern of findings will be discussed in the context of discrete emotions theories.

